# Users’ perspectives of key factors to implementing electronic health records in Canada: a Delphi study

**DOI:** 10.1186/1472-6947-12-105

**Published:** 2012-09-11

**Authors:** Carrie Anna McGinn, Marie-Pierre Gagnon, Nicola Shaw, Claude Sicotte, Luc Mathieu, Yvan Leduc, Sonya Grenier, Julie Duplantie, Anis Ben Abdeljelil, France Légaré

**Affiliations:** 1 Institut de réadaptation en déficience physique de Québec, Québec, Canada; 2 Research Centre of the Centre hospitalier universitaire de Québec, Québec, Canada; 3 Faculty of Nursing, Université Laval, Québec, Canada; 4 Health Informatics Institute, Algoma University, Algoma, Sault-Sainte-Marie, Canada; 5 Department of Health Management, Université de Montréal, Montréal, Canada; 6 Department of Nursing, Université de Sherbrooke, Sherbrooke, Canada; 7 Department of Social and Preventive Medicine, Université Laval, Québec, Canada; 8 Department of Family and Emergency Medicine, Université Laval, Québec, Canada

**Keywords:** Delphi technique, Adoption factors, Implementation factors, Electronic health record, Health information technology, Health communication technology, Medical informatics

## Abstract

**Background:**

Interoperable electronic health record (EHR) solutions are currently being implemented in Canada, as in many other countries. Understanding EHR users’ perspectives is key to the success of EHR implementation projects. This Delphi study aimed to assess in the Canadian context the applicability, the importance, and the priority of pre-identified factors from a previous mixed-methods systematic review of international literature.

**Methods:**

A three-round Delphi study was held with representatives of 4 Canadian EHR user groups defined as partners of the implementation process who use or are expected to use EHR in their everyday activity. These groups are: non-physician healthcare professionals, health information professionals, managers, and physicians. Four bilingual online questionnaire versions were developed from factors identified by the systematic review. Participants were asked to rate the applicability and the importance of each factor. The main outcome measures were consensus and priority. Consensus was defined a priori as strong (≥ 75%) or moderate (≥ 60-74%) according to user groups’ level of agreement on applicability and importance, partial (≥ 60%) when participants agreed only on applicability or importance, or as no consensus (< 60%). Priority for decision-making was defined as factors with strong consensus with scores of 4 or 5 on a five-point Likert scale for applicability and importance.

**Results:**

Three Delphi rounds were completed by 64 participants. Levels of consensus of 100%, 64%, 64%, and 44% were attained on factors submitted to non-physician healthcare professionals, health information professionals, managers, and physicians, respectively. While agreement between and within user groups varied, key factors were prioritized if they were classified as strong (≥ 75% from questionnaire answers of user groups), for decision-making concerning EHR implementation. The10 factors that were prioritized are perceived usefulness, productivity, motivation, participation of end-users in the implementation strategy, patient and health professional interaction, lack of time and workload, resources availability, management, outcome expectancy, and interoperability.

**Conclusions:**

Amongst all factors influencing EHR implementation identified in a previous systematic review, ten were prioritized through this Delphi study. The varying levels of agreement between and within user groups could mean that users’ perspectives of each factor are complex and that each user group has unique professional priorities and roles in the EHR implementation process. As more EHR implementations in Canada are completed it will be possible to corroborate this preliminary result with a larger population of EHR users.

## Background

### Electronic health records

There is currently worldwide interest in the potential of electronic health record (EHR) to reduce healthcare costs and improve significantly the quality of healthcare provided [1]. EHR programs are perceived as an opportunity to improve the health sector fundamentally. Nevertheless, these programs are complex and costly [2]. Deutsch and colleagues evaluated EHR programs in five countries (England, Germany, Canada, Denmark, and Australia) and found five critical areas for successful implementation: acceptance and change management, demonstration of benefits and funding, project management, health policy-related goals and implementation strategy [2].

EHRs differ from electronic medical records (EMRs) and personal health records (PHRs) most notably on the basis of the completeness of the information the record contains and the designated custodian of the information [3]. A PHR is often described as being a complete or partial health record under the custodianship of a person(s) (e.g. a patient or family member) that holds all or a portion of the relevant health information about that person over their lifetime. This is a person-centric health record. An EMR may be characterized as a partial health record under the custodianship of a health care provider(s) that holds a portion of the relevant health information about a person over their lifetime. This is often described as a provider-centric or health organization-centric health record of a person. In contrast, an EHR may be defined as a complete health record under the custodianship of a healthcare provider(s) that holds all relevant health information about a person over their lifetime. This is often described as a person-centric health record, which can be used by many approved healthcare providers or health care organizations.

In Canada, a network of interoperable electronic health record (EHR) network is however currently being implemented. Canada Health Infoway was created by Canada’s First Ministers in 2001 with the goal to foster and accelerate the building of a pan-Canadian electronic health record network which will manage Canadians’ health information. According to Canada Health Infoway, EHR solutions will link clinics, hospitals, pharmacies and other points of care. They hold the promise of helping to improve Canadians’ access to health services, enhance the quality of care and patient safety, and help the health care system becoming more efficient [4].

Recent systematic reviews show that EMR/EHR systems have not yet demonstrated clinical and economic benefits [1][5][6]. Other recent results indicate that computerized clinical decision support systems (CCDSSs), a feature that is often linked to the EHR, inconsistently improved process of care measures and seldom improved patient outcomes. Lack of clear patient benefit and lack of data on harms and costs precluded a recommendation to adopt CCDSSs for drug therapy management [7]. Moreover, it is possible that harms may neutralize many of the benefits expected from EMR, EHR or CCDSS [7]. The vision of Canada Health Infoway is to provide a secure and private EHR lifetime record of health history and care within the health system, available to authorized health providers and individuals. It is believed that EHRs will facilitate the sharing of data across the continuum of care, the healthcare delivery organizations, and geographical areas [8]. The Canadian Medical Association has called for government investment in health information systems such as EHRs with the goal of improving patient outcomes and system efficiency [9,10]. Nevertheless, the Canadian healthcare system remains paper-laden and EHR implementation lags behind many other industrial countries [11-14].

EHR implementation involves many user groups. In this study, users are defined as partners of the EHR implementation process and use or are expected to use EHRs in their everyday activities. A very recent Canadian study conclude that relatively few differences in perceptions about EHR system adoption and use exist between physicians already using such systems and those not yet using the systems [15]. A recent qualitative study among key stakeholders from various groups in Canada (provincial and regional representatives, health care professionals, public health agents, and vendors) reports important achievements and barriers to EHR implementation, but this study does not differentiate the views between these stakeholder groups [16]. This paper presents the findings of a Delphi study which explored the perspectives of different actual or potential EHR users in Canada. Achieving consensus among users on factors influencing the successful implementation of EHR through the Delphi technique appears as an important first step given the current lack of empirical evidence to inform decision-making on possible effective implementation strategies in the Canadian healthcare system. These preliminary data could be used as the basis for empirical study of effective EHR implementation strategies.

### The Delphi technique

The Delphi technique is based on a structured process for collecting and distilling knowledge from a group of experts by means of a series of questionnaires interspersed with controlled opinion feedback [17-19]. It is considered to be a strong methodology for achieving a rigorous consensus of experts on a specific theme. There is no consensus on the panel size for Delphi studies. As shown by Akins, Tolson & Cole (2005), reliable outcomes could be obtained with a relatively small Delphi panel of experts with similar training and general understanding in the field of interest [20], However generalizability of the results may consequently be reduced. Although some degree of interpretation and flexibility have been observed, a classic Delphi survey follows a set of procedures reflecting behavioural and statistical processes [19]. Three rounds of questionnaires are typically sent to a chosen panel of experts. However, the number of rounds may vary.

The main advantage of the Delphi technique is the achievement of consensus in a given area which is uncertain or lacks empirical evidence. This type of study is recommended for obtaining opinions from experts who live and work in different geographic regions and settings [19]. The feedback between rounds is interesting in itself given that it can be highly motivating and educational for the participants. Finally, the anonymity of the Delphi technique also encourages open and honest feedback among experts [19].

### Findings of the systematic review

The systematic review, covering a period from 1999 to 2009, was conducted on nine electronic databases (PubMed, EMBASE, CINAHL, Business Source Premier, Science Citation Index, Social Sciences Citation Index, Cochrane Library, ABI/Inform, and PsychINFO). The search strategy, developed by an information specialist, is available upon request. The key question was: “What can limit or contribute to the success of EHR implementation projects according to each group of EHR users?”. Studies were included if they reported on users’ perceived barriers and facilitators to EHR implementation, in healthcare settings comparable to Canada. Studies with an empirical study design were included [21]. The systematic review identified four key EHR user groups: physicians, other non-physician health professionals, healthcare managers, and patients. The findings show that the most frequent implementation factors common to all user groups were: design and technical concerns, ease of use, interoperability, privacy and security, costs, productivity, familiarity and ability with EHR, motivation to use EHR, patient and health professional interaction, and workload and lack of time. Each user group also identified factors specific to their professional and individual priorities. More details can be found in the article presenting this study [21].

### Study objectives

This Delphi study aimed to assess the applicability, the importance, and the priority of the results of the systematic review of international literature concerning users’ perspectives of the factors influencing EHR implementation [21,22]. It was driven by the desire to answer this question: What findings of the systematic review are applicable to the Canadian context and which factors are prioritized by each user group for future decision making regarding EHR implementation in Canada?

## Methods

We conducted a Delphi study among Canadian representatives of actual or potential EHR users to confirm the findings of the systematic review and to prioritize the key barriers and facilitating factors for EHR implementation in Canada.

### Participants

The study participants are Canadian representatives of actual or potential EHR users, or groups who use or are expected to use EHR. They are subdivided into four groups: physicians, patients, managers and non-physician health [23-25]. In order to better reflect the reality of the Canadian healthcare system, we subdivided non-physician health professionals in two groups: non-physician healthcare professionals and health information professionals. Non-physician healthcare professionals are nurses, physician assistants and other non-physician providers. Health information professionals (HIP) are defined as people providing leadership in all aspects of clinical information management at both the micro and macro levels. At the micro (or individual record level), HIP professionals support data collection, use, access and disclosure, to the retention and destruction of health information regardless of format. HIPs perform qualitative analysis on the documentation within the health record and are responsible for the security of health records. HIPs are advocates of the individual’s right to private, secure and confidential health information. At the macro (or aggregate data level), HIPs deal with the information through the health system, analyze statistics, manage complex information systems including registries and work with public, private and key stakeholders in understanding and using health data to improve the health of Canadians [26].

A purposive sampling method was used to recruit participants from all Canadian provinces and territories. More than 20 national and provincial e-health and healthcare professional associations, organizations and interest groups, such as the Canada Health Infoway Clinician Peer Support Network, were asked to collaborate through forwarding our study recruitment email to their members (see the full list in Additional file 1). Experts who were known actual or potential users of Canadian EHR systems were purposefully selected and also invited by telephone and email to join the study. Other potential key informants were identified through a snowball sampling technique. Interested participants were directed to the study website to register their contact information and detail their EHR experience.

Our selection criteria aimed to ensure an adequate breadth of expertise and representation of various geographical contexts. Eligible participants were required to have professional experience related to Canadian EHRs. Because there are few fully interoperable EHR projects in Canada, “EHR experience” was broadly defined as being knowledgeable about the electronic organization of patient information, including a full or partial computerized record of a person's medical history, or just certain records, such as laboratory or diagnostic testing results. Participants also needed to belong to one of the five EHR user groups (physicians, non-physician healthcare professionals, health information professionals, healthcare managers and patients), have a valid email address and access to the internet, and speak English or French. As required by the ethics committee, patient participants were also required to hold an official position of “patient representative” within a health association or organization.

### Ethical consideration

The Delphi study participants were given specific consent forms presenting research objectives and information about research implications. They were informed that their participation in the study was entirely voluntary and that they implicitly consented to participate by completing the first round of the electronic Delphi study. Ethics approval for the study protocol was received from the Research Ethics Board of the Centre Hospitalier Universitaire de Québec (approved January 23, 2009; ethics number 5-08-12-06).

### Design of the Delphi study

Based on factors identified for each users group in the systematic review [21] we designed a specific questionnaire for each actual or potential user group. The questionnaire proposed a number of questions that corresponded to the most frequent factors found for each user group in the literature review [21]. In order to limit the length of questionnaires we decided to select the factors that were mentioned by at least three studies, or exceptionally by two studies if one of them was conducted in Canada. Most studies included in the systematic review did not make a distinction between non-physician healthcare professionals and health information professionals. Consequently, it was then not possible to extract the factors specific to each non-physician health professional group. Healthcare professionals and health information professionals received the same questionnaire version (14 factors listed in Additional file 2), but each group was analysed separately because their answers were different. Then, four bilingual (English and French) online questionnaire versions, pertaining to each user group (physicians, non-physician health professionals, managers and patients) were developed (see complete questionnaires presented in Additional files 3, 2, 4, and 5). Two bilingual members of the research team validated the translation. The questionnaires were pre-tested by members of the research team to assess the clarity of the questions, the instructions and the format. Physicians and managers received questionnaire versions with respectively, 18 and 11 factors. Using a five-point Likert scale (where 1 indicated strong disagreement and 5, strong agreement), participants rated each questionnaire statement for its *applicability* to the participant’s specific EHR implementation context and its *importance* for decision-making regarding EHR implementation in Canada. Participants were also invited to leave written comments after each answer and general comments at the end of the questionnaire. The Delphi study was conducted during a 10 day period in March 2010, using a dedicated website developed for the purpose of this study.

For each Delphi study round, participants were emailed a link to the appropriate questionnaire on the project website, and were allotted 48 hours to complete the questionnaire. Email reminders to complete the questionnaire were sent after 24 hours to participants who had not yet replied. In the first round, participants were asked to simply rate their responses for each item. In the second and third rounds, distributions of participants’ answers to each item in the previous round were presented in percentage form. Participants were invited to take into consideration the other participants’ responses and reassess their answers in light of this new information. Questionnaire formats remained unchanged during the three study rounds and participants were not provided with reminders of their responses in the previous rounds. Individuals who did not complete a previous round were not invited to participate in following rounds.

### Analysis

Third round data was used for final analysis. As there is no existing definite criteria determining consensus in a Delphi study [27], we chose a priori consensus criteria based upon the research team’s previous work [28]. Consensus on a questionnaire factor was considered “strong” when at least 75% of participants reached an agreement on both the applicability and importance. We chose to consider these two criteria because this would indicate a priority for decision making [18]. “Moderate” consensus required 60% to 74% of participants to agree on both the applicability and the importance. “Partial” consensus was obtained when at least 60% of the participants reached consensus on only one aspect (applicability or importance) of a factor. Absence of consensus was determined when less than 60% of participants agreed on the applicability and the importance of a factor.

Percentile scores and interquartile range were used to calculate the level and strength of the consensus, respectively. To determine the level of consensus, tenth and twenty-fifth percentile scores were calculated. Tenth percentile scores indicate the lowest number on the Likert scale upon which at least 90% of participants agreed and 25th percentile scores indicate 75% agreement. Interquartile range, a measure of statistical dispersion, indicated the strength of the consensus, where 0 specifies a strong group consensus and 2 indicates dispersed responses. For example, in Table 1 physician consensus, the factor “confidence in EHR developer or vendor” (applicability) shows that according to 10^th^ percentile scores 90% of respondents responded either 2, 3,4 or 5 on the 5 point Likert scale, while 75% of respondents responded either 4 or 5. The interquartile range of 0 indicates strong group consensus.

**Table 1:** Physician consensus

**Strength of consensus**	**EHR implementation factor**	**Criteria**^**§**^**A = applicability I = importance**	Consensus*(Agreement in %)	**5-point Likert score**	**Percentile score**		**Inter- quartile range**
					**10th**	**25th**	
Strong	Confidence in EHR developer or vendor	A	≥ 75 %	4	2	4	0
		I	≥ 75 %	4	4	4	0
Moderate	Cost issues (start-up and maintenance)	A	≥ 75 %	4	3	4	0
		I	60-74 %	4	3	4	0
	Lack of time and workload (clinical tasks)	A	60-74 %	4	3	4	0
		I	60-74 %	4	4	4	1
Partial	Cost issues (return on investment)	A	60-74 %	4	1	2	2
		I	< 60 %	−	1	3	1
	Patient and health professional interaction	A	< 60 %	−	3	3	1
		I	60-74 %	4	3	3	1
	Lack of time and workload (EHR use)	A	60-74 %	4	2	4	0
		I	< 60 %	−	4	4	1
	Change in tasks	A	60-74 %	4	3	4	0
		I	< 60 %	−	3	4	1
	Choice of the EHR system	A	60-74 %	4	2	3	1
		I	< 60 %	−	2	3	1
None	Design and technical concerns	A	< 60 %	−	3	3	2
		I	< 60 %	−	3	3	2
	Privacy and security concerns (patient privacy)	A	< 60 %	−	1	2	2
		I	< 60 %	−	1	2	2
	Privacy and security concerns (professional confidentiality)	A	< 60 %	−	1	1	3
		I	< 60 %	−	1	3	2
	Quality standards	A	< 60 %	−	3	3	2
		I	< 60 %	−	3	3	2
	Productivity (loss of)	A	< 60 %	−	3	3	2
		I	< 60 %	−	3	4	1
	Practice size (small)	A	< 60 %	−	2	2	2
		I	< 60 %	−	2	3	2
	Practice size (large)	A	< 60 %	−	3	3	2
		I	< 60 %	−	3	3	2
	Physician salary status and reimbursement	A	< 60 %	−	2	2	2
		I	< 60 %	−	2	3	1
	Human resources (IT support, other)	A	< 60 %	−	1	3	2
		I	< 60 %	−	3	4	1
	Management	A	< 60 %	−	1	2	1
		I	< 60 %	−	2	2	1

^§^ A = applicability, I = importance.

* Agreement in %: ≥ 75 % agreement, 60-74 % agreement, < 60 % agreement.

Priority for decision-making concerning EHR implementation in Canada was determined based on the questionnaire items upon which 90% of participants considered both applicable and important (that is, factors scoring 4 or 5 for applicability and importance on the 5-point Likert scale). Tenth percentile scores were used to determine if the questionnaire item reached sufficient consensus. The analyses were performed using SAS software version 9.1.

## Results

### Characteristics of the participants

**Figure 1:**
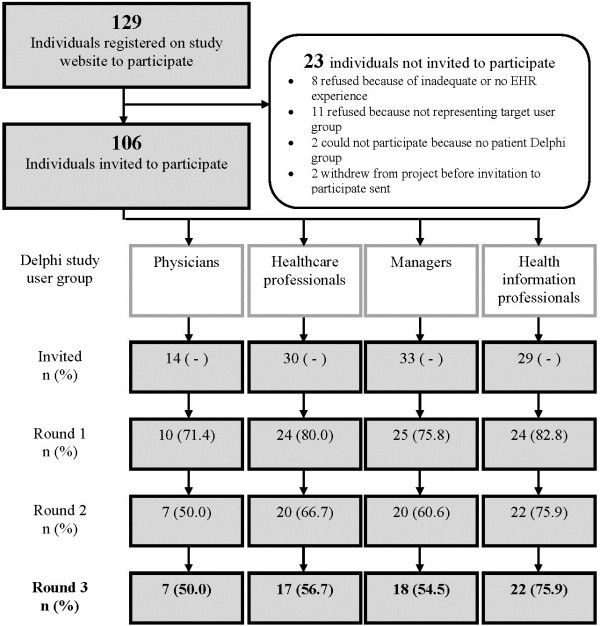
Flow diagram of the Delphi study participants.

Among 106 registered individuals invited to join one of the four Delphi study groups, 64 participants responded to all three rounds (Figure 1). The response rates were satisfactory and varied between 50% and 76% across the Delphi groups in the third round. We recruited a sufficient number of participants from the physician, other non-physician healthcare professionals, health information professionals, and managers groups, but not for patients (only two patient representatives completed the questionnaire, and this group was thus excluded). Respondents were mostly female (81%) and varied both in their professional occupations and area of residence (Table 2).

**Table 2:** Participant demographics, round 3 (n = 64)

**Characteristic**		**n (%)**
**Profession**		
	Health information professional	22 (34.4)
	Healthcare manager	18 (28.1)
	Nurse	15 (23.4)
	Occupational therapist	1 (1.6)
	Pharmacist	1 (1.6)
	Physician	7 (10.9)
**Sex**		
	Female	52 (81.2)
	Male	12 (18.8)
**Province or territory of residence**		
	Alberta	12 (18.8)
	British Columbia	6 (9.4)
	Manitoba	2 (3.1)
	Newfoundland	1 (1.6)
	Nova Scotia	4 (6.2)
	Ontario	28 (43.8)
	Quebec	7 (10.9)
	Saskatchewan	2 (3.1)
	Yukon	2 (3.1)

### Results of the Delphi study

Table 1 shows that nearly half (8/18) of the factors on the physicians’ questionnaire reached consensus on the applicability or importance: one item reached a strong consensus (≥ 75%), two items moderate consensus (≥ 60-74%), and five items partial consensus (≥ 60%). Healthcare professionals reached consensus on all factors of their questionnaire (Table 3). Health information professionals attained consensus on all but one of the 14 factors to a strong, moderate and partial level on 5, 4 and 4 factors, respectively (Table 4). Out of a total of 11 factors, managers achieved strong, moderate, and partial consensus on 2, 2, and 3 factors, respectively (Table 5).

**Table 3:** Healthcare professional consensus

**Strength of consensus**	**EHR implementation factor**	**Criteria**^**§**^**A = applicability I = importance**	Consensus*(Agreement in %)	**5-point Likert score**	**Percentile score**		**Inter-quartile range**
					**10th**	**25th**	
Strong	Perceived usefulness	A	≥ 75 %	5	4	5	0
		I	≥ 75 %	5	5	5	0
	Motivation	A	≥ 75 %	5	4	5	0
		I	≥ 75 %	5	4	5	0
	Patient and health professional interaction	A	≥ 75 %	4	4	4	0
		I	≥ 75 %	4	4	4	0
	Lack of time and workload (professional tasks)	A	≥ 75 %	5	4	5	0
		I	≥ 75 %	5	5	5	0
	Lack of time and workload (EHR use)	A	≥ 75 %	5	5	5	0
		I	≥ 75 %	5	5	5	0
	Resources available (additional)	A	≥ 75 %	5	4	5	0
		I	≥ 75 %	5	5	5	0
	Human resources (IT support, other)	A	≥ 75 %	5	3	5	0
		I	≥ 75 %	5	3	5	0
	Participation of end-users in implementation strategy	A	≥ 75 %	5	4	5	0
		I	≥ 75 %	5	5	5	0
Moderate	Productivity	A	60-74 %	5	4	4	1
		I	≥ 75 %	5	4	5	0
Partial	Design and technical concerns	A	< 60 %	−	4	4	1
		I	≥ 75 %	5	4	5	0
	Perceived ease of use	A	< 60 %		4	4	1
		I	≥ 75 %	−	4	5	0
	Privacy and security concerns	A	≥ 75 %	3	2	3	0
		I	< 60 %	−	3	3	2
	Outcome expectancy	A	< 60 %	−	4	4	1
		I	≥ 75 %	5	4	5	0
	Management	A	< 60 %	−	4	4	1
		I	≥ 75 %	5	4	5	0

^§^ A = applicability, I = importance.

* Agreement in %: ≥ 75 % agreement, 60-74 % agreement, < 60 % agreement.

**Table 4:** Health information professional consensus

**Strength of consensus**	**EHR implementation factor**	**Criteria**^**§**^**A = applicability I = importance**	Consensus*(Agreement in %)	**5-point Likert score**	**Percentile score**		**Inter- quartile range**
					**10th**	**25th**	
Strong	Perceived usefulness	A	≥ 75 %	5	4	5	0
		I	≥ 75 %	5	4	5	0
	Productivity/efficiency	A	≥ 75 %	5	4	5	0
		I	≥ 75 %	5	5	5	0
	Motivation	A	≥ 75 %	5	4	5	0
		I	≥ 75 %	5	4	5	0
	Management	A	≥ 75 %	5	5	5	0
		I	≥ 75 %	5	5	5	0
	Participation of end-users in implementation strategy	A	≥ 75 %	5	5	5	0
		I	≥ 75 %	5	5	5	0
Moderate	Design and technical concerns	A	60-74 %	5	3	4	1
		I	60-74 %	5	4	4	1
	Perceived ease of use	A	60-74 %	5	3	4	1
		I	60-74 %	5	4	4	1
	Resources available	A	≥ 75 %	5	4	5	0
		I	60-74 %	5	3	4	1
	Human resources (IT support, other)	A	60-74 %	5	3	4	1
		I	≥ 75 %	5	4	5	0
Partial	Privacy and security concerns	A	< 60 %	−	3	3	2
		I	60-74 %	5	4	4	1
	Outcome expectancy	A	< 60 %	−	4	4	1
		I	60-74 %	5	4	4	1
	Lack of time and workload (professional tasks)	A	< 60 %	−	4	4	1
		I	60-74 %	5	2	4	1
	Lack of time and workload (EHR use)	A	< 60 %	−	2	3	1
		I	≥ 75 %	5	3	5	0
None	Patient and health professional interaction	A	< 60 %	−	2	2	2
		I	< 60 %	−	2	3	2

^§^ A = applicability, I = importance.

* Agreement in %: ≥ 75 % agreement, 60-74 % agreement, < 60 % agreement.

**Table 5:** Manager consensus

**Strength of consensus**	**EHR implementation factor**	**Criteria**^**§**^**A = applicability I = importance**	Consensus*(Agreement in %)	**5-point Likert score**	**Percentile score**		**Inter-quartile range**
					**10th**	**25th**	
Strong	Interoperability	A	≥ 75 %	5	5	5	0
		I	≥ 75 %	5	5	5	0
	Outcome expectancy	A	≥ 75 %	5	5	5	0
		I	≥ 75 %	5	5	5	0
Moderate	Resources available	A	60-74 %	5	1	4	1
		I	≥ 75 %	5	4	5	0
	Training	A	60-74 %	5	1	3	2
		I	≥ 75 %	5	4	5	0
Partial	Cost issues	A	< 60 %	−	2	4	1
		I	≥ 75 %	5	4	5	0
	Human resources (IT support, other)	A	< 60 %	−	1	4	1
		I	≥ 75 %	5	4	5	0
	Choice of the system	A	< 60 %	−	2	3	2
		I	60-74 %	5	2	4	1
None	Privacy and security concerns (security of patient information)	A	< 60 %	−	1	3	1
		I	< 60 %	−	2	3	1
	Privacy and security concerns (patient privacy)	A	< 60 %	−	2	2	2
		I	< 60 %	−	2	4	1
	Familiarity, ability with EHR	A	< 60 %	−	1	1	1
		I	< 60 %	−	1	1	1
	Lack of time and workload	A	< 60 %	−	1	2	1
		I	< 60 %	−	1	2	3

^§^ A = applicability, I = importance.

* Agreement in %: ≥ 75 % agreement, 60-74 % agreement, < 60 % agreement.

**Figure 2:**
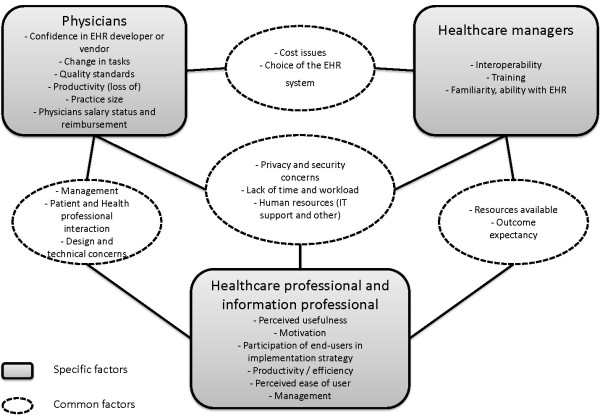
Common and specific EHR adoption factors among Delphi groups.

Comparison between all groups is limited, as each group received a different questionnaire adapted to reflect the findings of the systematic review [21] specific to each actual or potential user group. Figure 2 shows the interrelationship of questionnaire items. As health professionals and health information professionals received the same questionnaire, they are most easily compared and generally shared points of view for most questionnaire items. The three following factors were common to all questionnaires, though question format varied: “privacy and security concerns”, “lack of time and workload”, and “human resources”. Consensus for these three factors varied among all groups (Table 6).

**Table 6:** Comparison of similar EHR implementation factors among Delphi groups

**EHR implementation factor**	**User group**^§^(strength of consensus*)			
	**P**	**HP**	**HI**	**M**
Privacy and security concerns	None	Partial	Partial	None
Lack of time and workload	Moderate/Partial	Strong	Partial	None
Human resources (IT support, other)	None	Strong	Moderate	Partial

^§^ P = physicians, HP = healthcare professionals, HI = health information professionals, M = managers.

* Strong = ≥ 75 % of participants agree on the applicability and importance EHR implementation factor, Moderate = ≥ 60-74 % of participants agree on the applicability and importance EHR implementation factor, Partial = ≥ 60 % of participants agree on one aspect of an EHR implementation factor, either its applicability or importance, None = < 60 % of participants agree on the applicability and importance of an EHR implementation factor.

As indicated in the Additional files 3, 2, 4, and 5, each of the items was associated to an EHR implementation factor. Ten decision-making factors, specific to EHR implementation in Canada (Table 7), are among the elements of strong consensus for health professionals, health information professionals, and managers. These 10 factors are perceived usefulness, productivity, motivation, participation of end-users in the implementation strategy, patient and health professional interaction, lack of time and workload, resources availability, management, outcome expectancy, and interoperability. These factors were prioritized on the account that at least 90% of participants agreed with a Likert scale rating of 4 or 5. Because this criterion was not satisfied, we were unable to prioritize physicians’ responses.

**Table 7:** Prioritization of EHR implementation factors for decision-making, by EHR user group

	User group*		
**EHR implementation factor**	**Healthcare professionals(Agreement in %)**	**Health information professionals(Agreement in %)**	**Managers(Agreement in %)**
Perceived usefulness	≥ 75 %	≥ 75 %	
Productivity	≥ 75 %	≥ 75 %	
Motivation	≥ 75 %	≥ 75 %	
Patient and health professional interaction	≥ 75 %		
Lack of time and workload (professional tasks & EHR use)	≥ 75 %		
Resources available	≥ 75 %		
Participation of end-users in implementation	≥ 75 %	≥ 75 %	
Management		≥ 75 %	
Outcome expectancy			≥ 75 %
Interoperability			≥ 75 %

*Physicians are not presented in this table as this user group did not meet prioritization criteria.

* Agreement in %: ≥ 75 % agreement.

## Discussion

This study aimed to gather this first-hand knowledge held by actual and potential Canadian EHR users. Using the Delphi method, we compared the implementation experiences in the Canadian context with the known factors found in the scientific literature. Our findings show that, overall, many of the factors found in the scientific literature are also applicable to the Canadian context. However the varying levels of agreement between and within user groups could mean that each group has unique professional priorities in the EHR implementation process.

This study is addressing an issue of great relevance in the current Canadian health care context. Notably, key factors to EHR implementation were considered of the highest priority in a recently published report investigating gaps in knowledge, research, and research capacity regarding EHRs in the Canadian primary health care [29].

However, the results of this study must be interpreted cautiously due to some limitations. In fact, the major flaw in this kind of study is that it relies on the perception of participant on what might potentially influence implementation success without actual evidence from real implementation. We would like to invite EHR implementation researchers to further explore this list of priorities in order to validate them in diverse implementation contexts. Also, despite exceptional recruitment efforts, only two representatives of patient groups volunteered to take part in the study. Consequently, the patient Delphi study was cancelled creating an important gap to our findings. As mentioned above, the small survey sample may reduce generalizability of the results. Also, the 50% participation rate of physicians caused limited prioritization from this group. As well, a bias introduced by a participation dominated by women could occur. Although profession is an important factor contributing to group consensus, other factors such as age, work setting, and specific type of EHR experience are influences that were not considered in this Delphi study. Consequently, the factors presented might have not been exhaustive of the respondents’ experiences and important factors may have been overlooked. Secondly, we do not know if the participants that dropped out of the study and the resulting missing data could represent a bias for the degree of consensus. Finally, results of this study are based upon the informed opinions of the participants, and results should be interpreted appropriately as the well-founding of such opinions cannot be verified.

The three factors common to all user groups reached different levels of consensus (see Table 6). Firstly, physicians and managers disagreed while health professionals and health information professionals partially agreed on the applicability or the importance of “Privacy and security concerns”. Our research question was centered on “which” factors were a priority. The limited consensus reached on certain aspects of the EHR implementation raises the question of “why” this is. Range in practice size or differences in professional settings could be an explanation in some instances, however only a dedicated study can answer this question. Secondly, perceptions toward “Lack of time and workload” appear to be user specific as indicated by strong, moderate, partial or no consensus for health professionals, physicians, health information professionals, and managers, respectively. Thirdly, “Human resources” presents a different consensus pattern in which health professional reached a strong consensus whereas health information professionals reached a moderate consensus. Additionally, physicians disagreed while managers partially agreed on the applicability or the importance of “Human resources”. Consensual factors of applicability and importance identified in our study for each user group should guide future research. We compared the Delphi study results with the factors specifically mentioned by the Canadian studies included in the systematic review [30-35]. Firstly, among the 17 studies included in our systematic review pertaining to physicians, the factor “confidence in the EHR developer” was mentioned only twice (including one Canadian study [33]). However, Canadian physicians reached a strong consensus about this factor in our Delphi study, indicating that this is an issue of particular relevance that should be explored in further research. Furthermore, while one Canadian study identified fee-for-service payment of Canadian physicians as a barrier to EHR implementation [33], our physician participants did not reach consensus on this subject. Updated research by the same authors confirms our findings, suggesting that this remuneration approach does not hinder EHR implementation more than other forms of payment [36]. Our findings concerning healthcare professionals are congruent with those of a recently published Canadian case study [37]. This study identified facilitating EHR implementation factors such as perceived usefulness, motivation, effective onsite technical support and management, and barriers such as concerns about privacy and security. Participants in our Delphi study reached consensus on all these factors. Moreover, while no Canadian study included in our systematic review mentioned the factors “lack of time and workload” or “resources available”, we found that Canadian healthcare professionals perceive these factors as important barriers to EHR implementation. A recent Canadian report [38] confirms our findings, indicating that significant labour and skills shortages among health professionals experienced with electronic health information systems are likely to constrain the successful implementation of such systems in Canada.

## Conclusion

This study provides some key findings that corroborate and expand current knowledge of the factors influencing EHR implementation in Canada. We conclude that the perspectives and priorities concerning the barriers and facilitating factors to EHR implementation in Canada could vary greatly between and within user groups and present a challenge to successful implementation of EHR programs [39].

In summary, we would like to invite EHR implementation researchers to further explore the list of priority factors specific to Canadian user groups that were identified in this Delphi study. Although important recruitment efforts have been made, it was not possible to recruit sufficient patient representatives and the patient Delphi study was thus cancelled. This limitation should be taken into account in future research.

### Summary table

#### What was already known on this subject

There is currently a strong focus worldwide on the potential of EHRs to reduce healthcare costs and significantly improve the quality of healthcare provided.

The implementation of EHR programs is complex and involves many user groups (e.g. physicians, healthcare professionals, health information professionals, managers, and patients).

Understanding users’ perspectives of EHR implementation is essential to the effective implementation of EHRs and to the successful integration of EHRs into the healthcare system.

#### What this study added to our knowledge

Amongst all barriers and facilitators to EHR implementation identified in a previous systematic review, ten were prioritized in the context of the Canadian healthcare system through this Delphi study.

Users’ perspectives of barriers and facilitators are complex and each user group has unique professional priorities and roles in the EHR implementation process.

We recommend that decision-makers integrate the perspectives specific to each user group, and consider the elements of consensus that emerged from this Delphi study, when implementing future EHR projects.

## Competing interests

All authors declare that they have no competing interests.

## Authors’ contributions

All authors contributed to the design of the study. CAM coordinated the Delphi study and drafted the article, which was critically revised by MPG, FL, CS, NS, SG, and ABA. All authors approved the final manuscript submitted for publication. MPG is the guarantor for the study. All authors had full access to all of the data (including statistical reports and tables) in the study and can take responsibility for the integrity of the data and the accuracy of the data analysis.

## Pre-publication history

The pre-publication history for this paper can be accessed here:

http://www.biomedcentral.com/1472-6947/12/105/prepub

## Supplementary Material

Additional file 1 Associations, organisations, and interest groups solicited to participate in the Delphi study. Click here for file

Additional file 2 Healthcare professional and health information professional questionnaire. Click here for file

Additional file 3 Physician questionnaire. Click here for file

Additional file 4 Manager questionnaire. Click here for file

Additional file 5 Patient questionnaire. Click here for file
