# Supplementary material for: Users’ perspectives of key factors to implementing electronic health records in Canada: a Delphi study
Source: BMC Med Inform Decis Mak. 2012 Sep 11;12:105. doi: 10.1186/1472-6947-12-105 (PMC3470948; doi:10.1186/1472-6947-12-105)
Supplement: Additional file 1 — Associations, organisations, and interest groups solicited to participate in the Delphi study. [file 1472-6947-12-105-S1.pdf]

Additional file 1: Associations, organisations, and interest groups solicited to participate in the Delphi study

Alberta Netcare
Association québécoise des infirmières et infirmiers en systèmes et technologies de l'information
Association québécoise des archivistes médicales
British Colombia eHealth branch
Canadian Health Information Management Association
Canada's Health Informatics Association
Canada Health Infoway – Clinician Peer Support Network
Canadian Institute for Health Information
Canadian Medical Association
Canadian Patient Safety Institute
Canadian Pharmacists Association
Canadian Society for Telehealth
Cientis Technologies
Dossier de Santé du Québec - *Comité consultatif clinique du projet*
Dossier de Santé du Québec - *Réseau du soutien et validation par les pairs (RSVP)*
eHealth Ontario
Manitoba e-Health Program
National ePharmacy Task Force
Nova Scotia Nursing Informatics Group
Ontario Patient Relations Association
Registered Nurses' Association of Ontario
Saskatchewan Health Information Solutions Centre
Société québécoise d'informatique biomédicale et de la santé
