# Supplementary material for: Users’ perspectives of key factors to implementing electronic health records in Canada: a Delphi study
Source: BMC Med Inform Decis Mak. 2012 Sep 11;12:105. doi: 10.1186/1472-6947-12-105 (PMC3470948; doi:10.1186/1472-6947-12-105)
Supplement: Additional file 2 — Healthcare professional and health information professional questionnaire. [file 1472-6947-12-105-S2.pdf]

Additional file 3: Healthcare professional and health information professional questionnaire

Items	EHR implementation factor
1) The technical limitations of EHRs (for example, slow computer system speed) are a barrier to EHR implementation.	Design and technical concerns
2) Healthcare professionals' perception of the advantages and the usefulness of EHRs is a facilitator to EHR implementation.	Perceived usefulness
3) Healthcare professionals' perception that EHRs are easy to use is a facilitator to EHR implementation.	Perceived ease of use
4) Healthcare professionals' concern that EHR use may compromise patient privacy is a barrier to EHR implementation.	Privacy and security concerns (patient privacy)
5) Healthcare professional's perception that EHRs contribute to improving productivity and efficiency is a facilitator to EHR implementation.	Productivity
6) Healthcare professionals' perception that EHRs positively influence healthcare services is a facilitator to EHR implementation.	Outcome expectancy (influence on healthcare services)
7) Healthcare professionals' motivation to use EHRs is a facilitator to EHR implementation.	Motivation to use EHR
8) Healthcare professionals' concern that EHRs may compromise their interaction with the patient is a barrier to EHR implementation.	Patient and health professional interaction
9) The heavy workload of many healthcare professionals is a barrier to EHR implementation.	Lack of time and workload (professional tasks)
10) Healthcare professionals' concern about EHR use being time-consuming is a barrier to EHR implementation.	Lack of time and workload (EHR use)
11) Lack of human resources (for example, extra staff) to support EHR implementation is a barrier to EHR implementation.	Resources available (for implementation)
12) Lack of technical support is a barrier to EHR implementation.	Human resources (IT support)
13) Effective management of the transition period to EHRs (for example, fair distribution of tasks among staff) is a facilitator to EHR implementation..	Management (strategic plan to implement EHR)
14) Participation of healthcare professionals in the EHR implementation process is a facilitator to EHR implementation.	Participation of end-users in the implementation strategy
