# Supplementary material for: Users’ perspectives of key factors to implementing electronic health records in Canada: a Delphi study
Source: BMC Med Inform Decis Mak. 2012 Sep 11;12:105. doi: 10.1186/1472-6947-12-105 (PMC3470948; doi:10.1186/1472-6947-12-105)
Supplement: Additional file 3 — Physician questionnaire. [file 1472-6947-12-105-S3.pdf]

Additional file 2: Physician questionnaire

Items	EHR implementation factor
1) The technical limitations of EHRs (for example, slow computer system speed) are a barrier to EHR implementation.	Design and technical concerns
2) Physicians' concerns that EHR use may compromise patient privacy are a barrier to EHR implementation.	Privacy and security concerns (patient privacy)
3) Physicians' concerns that EHR use may compromise the confidentiality of their professional work are a barrier to EHR implementation.	Privacy and security concerns (professional confidentiality)
4) Uniformity of standards for EHR systems is a facilitator to EHR implementation.	Quality standards
5) Physicians' concerns about the high start-up and ongoing maintenance costs associated with EHR implementation and use are a barrier to EHR implementation.	Cost issues (start-up and maintenance)
6) Physicians' concerns of an inadequate return on investment are a barrier to EHR implementation.	Cost issues (return on investment)
7) Physicians' concerns about loss of productivity during transition to EHRs are a barrier to EHR implementation.	Productivity (loss of)
8) Physicians' lack of confidence in EHR vendors is a barrier to EHR implementation. For example, concerns that an EHR vendor may provide inadequate support or go out of business.	Confidence in EHR developer or vendor
9) Physicians' concerns that EHRs may compromise physician-patient interaction are a barrier to EHR implementation.	Patient and health professional interaction
10) A solo or small practice size is a barrier to EHR implementation.	Practice size (small)
11) A large practice size is a facilitator to EHR implementation.	Practice size (large)
12) The fee-for-service payment of Canadian physicians is a barrier to EHR implementation, as time used implementing EHRs reduces revenue generated.	Physician salary status and reimbursement
13) Physicians' lack of time to acquire, implement and learn to use EHRs is a barrier to EHR implementation.	Lack of time and workload (EHR use)
14) Physicians' concerns that EHR implementation would take time away from their clinical tasks is a barrier to EHR implementation.	Lack of time and workload (clinical tasks)
15) Physicians' concerns about taking on additional tasks and responsibilities that are not presently considered their responsibility are a barrier to EHR implementation.	Change in tasks
16) Lack of technical support is a barrier to EHR implementation.	Human resources (IT support)
17) Physicians' concern that management may use EHRs as a way to control their actions is a barrier to EHR implementation.	Management (strategic plan to implement EHRs)
18) Standardization of the EHR acquisition process is a facilitator to EHR implementation.	Choice of the EHR system
