# Supplementary material for: Users’ perspectives of key factors to implementing electronic health records in Canada: a Delphi study
Source: BMC Med Inform Decis Mak. 2012 Sep 11;12:105. doi: 10.1186/1472-6947-12-105 (PMC3470948; doi:10.1186/1472-6947-12-105)
Supplement: Additional file 4 — Manager questionnaire. [file 1472-6947-12-105-S4.pdf]

Additional file 4: Manager questionnaire

Items	EHR implementation factor
1) Adequate electronic data exchange among different clinical systems (system interoperability) is a facilitator to EHR implementation.	Interoperability
2) Managers' concern that EHR use may compromise the security of patient information is a barrier to EHR implementation.	Privacy and security concerns (security of patient information)
3) Managers' concern that EHR use may compromise patient privacy is a barrier to EHR implementation.	Privacy and security concerns (patient privacy)
4) Managers' concern about the high costs associated with EHR implementation and use is a barrier to EHR implementation.	Cost issues
5) Managers' concern about patients' lack of familiarity with computers is a barrier to EHR implementation.	Familiarity, ability with EHR
6) Managers' perception of the benefits of EHRs (for example, that EHRs will improve information sharing) is a facilitator to EHR implementation.	Outcome expectancy (benefits of EHR)
7) Managers' concern about EHR use increasing physician workload is a barrier to EHR implementation.	Lack of time and workload
8) Lack of technical support is a barrier to EHR implementation.	Human resources (IT support)
9) Lack of human resources (for example, extra staff) to support EHR implementation is a barrier to EHR implementation.	Resources available (for implementation)
10) Lack of employee training is a barrier to EHR implementation.	Training
11) Managers' concern about choosing an inappropriate EHR system is a barrier to EHR implementation.	Choice of the EHR system
