# Supplementary material for: Users’ perspectives of key factors to implementing electronic health records in Canada: a Delphi study
Source: BMC Med Inform Decis Mak. 2012 Sep 11;12:105. doi: 10.1186/1472-6947-12-105 (PMC3470948; doi:10.1186/1472-6947-12-105)
Supplement: Additional file 5 — Patient questionnaire. [file 1472-6947-12-105-S5.pdf]

Additional file 5: Patient questionnaire

Items	EHR implementation factor
1) Patients' perception of the advantages and the usefulness of EHRs (for example, that EHRs will improve communication with their healthcare provider) is a facilitator to EHR implementation.	Perceived usefulness
2) Patients' concern about EHR systems being less reliable than paper based information systems (for example, concerns about data loss) is a barrier to EHR implementation.	System reliability or dependability
3) Patients' perception that EHRs will improve the accuracy of their health record is a facilitator to EHR implementation.	Accuracy
4) Patients' concern that EHR use may compromise the confidentiality of their health information is a barrier to EHR implementation.	Privacy and security concerns (confidentiality)
5) Patients' concern that EHR use may compromise the security of their health information is a barrier to EHR implementation.	Privacy and security concerns (security)
6) Patients' perception that the information contained in their EHR is easy to understand is a facilitator to EHR implementation.	Content appropriate to the user
7) Patients' perception that EHRs contribute to improving the productivity and efficiency of clinical work is a facilitator to EHR implementation.	Productivity
8) Patients' lack of familiarity with computers is a barrier to EHR implementation.	Familiarity, ability with EHR
9) Patients' perception that the advantages of EHRs outweigh the disadvantages of EHRs is a facilitator to EHR implementation.	Risk-benefit equation
10) Patients' perception that EHRs may aid in taking a more active role in managing their own health is a facilitator to EHR implementation.	Autonomy
11) Patients' motivation to use EHRs is a facilitator to EHR implementation.	Motivation to use EHR
